# Caspase-independent programmed cell death triggers Ca_2_PO_4_ deposition in an *in vitro* model of nephrocalcinosis

**DOI:** 10.1042/BSR20171228

**Published:** 2018-01-17

**Authors:** Giovanna Priante, Federica Quaggio, Lisa Gianesello, Monica Ceol, Rosalba Cristofaro, Liliana Terrin, Claudio Furlan, Dorella Del Prete, Franca Anglani

**Affiliations:** 1 Department of Medicine – DIMED, Kidney Histomorphology and Molecular Biology Laboratory, Clinical Nephrology, University of Padua, Padua, Italy; 2 Center for Laboratory Analyses and Certification Services (CEASC), University of Padua, Padua, Italy

**Keywords:** caspases, cell death, GDNF, nephrocalcinosis, osteogenesis, renal epithelial cell

## Abstract

Nephrocalcinosis involves the deposition of microscopic crystals in the tubular lumen or interstitium. While the clinical, biochemical, and genetic aspects of the diseases causing nephrocalcinosis have been elucidated, little is known about the cellular events in this calcification process. We previously reported a phenomenon involving the spontaneous formation of Ca_2_PO_4_ nodules in primary papillary renal cells from a patient with medullary nephrocalcinosis harboring a rare glial cell-derived neurotrophic factor (*GDNF*) gene variant. We also demonstrated that cultivating *GDNF*-silenced human kidney-2 (HK-2) cells in osteogenic conditions for 15 days triggered Ca_2_PO_4_ deposits. Given the reportedly close relationship between cell death and pathological calcification, aim of the present study was to investigate whether apoptosis is involved in the calcification of *GDNF*-silenced HK-2 cells under osteogenic conditions. Silenced and control cells were cultured in standard and osteogenic medium for 1, 5, and 15 days, and any Ca_2_PO_4_ deposition was identified by means of von Kossa staining and environmental SEM (ESEM) analyses. Based on the results of annexin V and propidium iodide (PI) analysis, and terminal deoxynucleotidyl transferase dUTP-biotin nick end labeling (TUNEL) assay, the silenced cells in the osteogenic medium showed a significant increase in the percentage of cells in the late phase of apoptosis and an increased Ca_2_PO_4_ deposition at 15 days. The results of quantitative real-time PCR (qRT-PCR) of *BAX* and *BCL2*, and in-cell Western analysis of caspases indicated that the cell death process was independent of caspase-3, -6, -7, and -9 activation, however. Using this model, we provide evidence of caspase-independent cell death triggering the calcification process in *GDNF*-silenced HK-2 cells.

## Introduction

Many *in vitro* and *in vivo* studies on the mechanisms underlying calcium nephrolithiasis have provided evidence of a condition frequently associated with nephrocalcinosis, which involved the deposition of microscopic crystals in the tubular lumen (intratubular nephrocalcinosis) or interstitium (interstitial nephrocalcinosis) [1–3]. While the clinical, biochemical, and genetic aspects of the diseases causing nephrocalcinosis have been thoroughly elucidated, little is known about the specific cellular events involved in this calcification process. The most accredited hypothesis to explain the onset of interstitial nephrocalcinosis is purely physicochemical and related to spontaneous Ca_2_PO_4_ crystallization in the interstitium due to calcium and phosphate oversaturation in this milieu [4,5]. Exactly how the tubulo-interstitial cells respond to the influx of these potentially precipitating ions is still unknown. We were the first to suggest that nephrocalcinosis might be an osteogenic-like cell-driven process [6,7], and our previous studies provided the first evidence of human renal cells undergoing calcification under certain circumstances, such as glial cell-derived neurotrophic factor (*GDNF*) gene down-regulation, in much the same way as in vascular calcification [8]. We had the chance to observe spontaneous instances of this phenomenon in primary renal cells derived from a patient with medullary sponge kidney (MSK) and interstitial nephrocalcinosis who carried a *GDNF* gene mutation. We also demonstrated that, when exposed to an osteogenic medium, renal tubular human kidney-2 (HK-2) cells with a silenced *GDNF* expression were better able to produce Ca_2_PO_4_ deposits than control cells by shifting the osteonectin/osteopontin ratio in favor of osteonectin. This finding was the first indication of a role for GDNF in the tubular renal cell calcification process [8].

A fundamental question remaining to be answered concerns the cellular mechanisms by means of which *GDNF* down-regulation promotes the calcification process. The assumption explored in the present study was that down-regulated *GDNF* could favor cell death phenomena, and apoptosis in particular. The importance of cell death in pathological calcification has been well documented [9–12]. It has been claimed, for instance that chondrocyte-derived apoptotic bodies might contribute to the calcification of articular cartilage [10]. In advanced carotid atherosclerotic plaques, matrix vesicle-like structures derived from vascular smooth muscle cells (VSMCs) were found to contain high levels of BAX, a pro-apoptotic member of the BCL2 family, indicating that they may be remnants of apoptotic cells [11,12]. Apoptotic VSMC-derived matrix vesicle-like structures can also concentrate and crystallize calcium, triggering calcification [12–15]. These findings suggest that calcification may be initiated by apoptotic bodies in co-operation with matrix vesicles. It has long been known that pathological calcification follows necrosis in cardiovascular tissues and in the kidney [16–18]. In normal bone formation too, calcification is initiated in matrix vesicles, released from osteoblasts and hypertrophic chondrocytes, and facilitated by apoptotic bodies [19–23].

All these findings led to the idea that cell death could be important in initiating ectopic calcification in renal cells under certain conditions. To test our hypothesis, we used a previously adopted *in vitro* experimental model of down-regulated *GDNF* in HK-2 cells [8]. This enabled us to demonstrate that cell death can trigger the calcification process in renal tubular cells, and that *GDNF* down-regulation strongly facilitates this process. We thus confirmed the role of GDNF as an adaptive survival factor, and its alteration appears to have a key role in nephrocalcinosis. We also discovered that, in *GDNF*-silenced cells, death occurs in a programmed but caspase-independent manner.

## Experimental

### Cell culture

The human renal proximal tubular cell line HK-2 was purchased from American Type Culture Collection (ATCC) (CRL-2190™). HK-2 cells were maintained in a mixture of Ham’s F12 and Dulbecco’s modified Eagle’s growth medium (DMEM/F12; EuroClone, CelBio) supplemented with 10% heat-inactivated FBS (HI-FBS), 2 mM l-glutamine, 100 U/ml penicillin, and 100 μg/ml streptomycin (EuroClone, CelBio). Cells were grown in a humidified atmosphere of 5% CO_2_ and 95% air at 37°C. The cells were seeded at an appropriate cell density for different assays and left to grow to 80% confluence, then cell synchronization was performed routinely by incubating cells in serum-free medium for 24 h prior to each experiment. The cells were exposed to different experimental conditions. The growth medium was replaced every 2–3 days.

### *GDNF* knockdown in the HK-2 cell line

Our *in vitro* model of nephrocalcinosis was established by silencing *GDNF* in HK-2 cells. To obtain stable *GDNF*-silenced cells, five different shRNAs targetting human *GDNF* (NM_000514) purchased from Sigma–Aldrich were used. Each plasmid was separately transfected, and a sixth transfection was performed with all five plasmids concurrently, according to the manufacturer’s protocol (Mirus, Madison). Cells (1.5 × 10^5^ cells per well) were transfected with 3 μg of plasmid DNA using the TransIT-LT1 transfection reagent (Mirus, Madison). Negative control cells were transfected with an empty pRS plasmid vector without shRNA (TR20003), using the same amount of TransIT-LT1 transfection reagent. Transfected cells underwent several weeks of selection with 0.75 µg/ml puromycin (Sigma–Aldrich), and clones with different resistances were obtained from each 29mer shRNA targetting *GDNF* mRNA, as well as from the corresponding negative controls. *GDNF* mRNA expression was assessed in all the clones using quantitative real-time PCR (qRT-PCR), as described below. *GDNF* silencing was also assessed at protein level using immunocytochemistry (ICC) with a polyclonal GDNF antibody (Santa Cruz Biotechnology). Briefly, ICC staining was performed on HK-2 cells fixed with cold methanol for 5 min at room temperature (RT). The cells were then treated with 3% H_2_O_2_ in PBS (pH 7.4) for 15 min at RT to remove endogenous peroxidase activity, and incubated with 2% normal goat serum (Sigma–Aldrich) for 30 min at RT to prevent non-specific antibody binding. Samples were incubated with a rabbit antibody targetting GDNF (Santa Cruz Biotechnology) diluted 1:200 in PBS at 4°C overnight. Samples were then rinsed with PBS and treated with a DakoCytomation EnVision+System-HRP Labeled Polymer anti-rabbit antibody (DAKO Corporation) in a humidified chamber at RT for 30 min. Signals were visualized using the chromogen 3,3-diaminobenzidine-tetrachloride (DAB, DAKO), and the cells were counterstained with Hematoxylin. The specificity of the immunolabeling was confirmed in treated cells without the primary antibody. Slides were analyzed by Diaplan light microscope (Leitz). Images were acquired using a Micropublisher 5.0 RTV camera (Q Imaging). GDNF protein quantitation was performed by morphometric analysis.

Amongst the shRNA sequences, the one exhibiting the greatest degree of silencing was chosen for all subsequent experiments (shRNA 4E).

### Osteogenic culture of HK-2 cells

Wild-type (WT), negative control (cells transfected with an empty vector), and *GDNF*-silenced HK-2 (shRNA 4E) cells were cultured in six-well tissue culture plates (Falcon™ Polystyrene Microplates, Thermo Scientific) at a density of 4.5 × 10^4^ cells per well in commercially available osteogenic medium (NH OsteoDiff medium, Miltenyi Biotec) for 1, 5, or 15 days. Control conditions were established by culturing cells in DMEM/F12 medium. The osteogenic and the DMEM/F12 medium were both replaced every 2–3 days for up to 15 days. The osteogenic stimulation experiments were run twice.

### Detecting and quantitating calcification

#### Alkaline phosphatase staining

Histochemical staining for alkaline phosphatase (ALP), an enzyme involved in bone matrix mineralization and an early marker of committed osteogenic cells, was performed with a commercially available kit (SIGMA FAST BCIP/NBT, Sigma–Aldrich). Cells were seeded in chamber slides (4 × 10^3^ cells/well; Nunc Lab-Tek Chamber Slide system; eight wells on Permanox; Thermo Scientific). After incubation in standard or osteogenic media for 1, 5, or 15 days, cells were washed twice with PBS and then fixed with prechilled methanol for 10 min at RT. After the methanol was removed, cells were washed with deionized H_2_O, treated with SIGMA FAST BCIP/NBT substrate on the cell culture chamber slides, and agitated slowly on a plate shaker for 10 min at RT. The substrate solution was aspirated, and the slides were washed twice with deionized H_2_O. Slides were mounted in a glycerol and water solution, and analyzed under a Leica DMIL LED phase-contrast inverted microscope (Leica Microsystems). Images were acquired using a LEICA ICC50W camera. ALP positive signals in the cells were detected as an intense granular blue/purple stain and were quantitated by morphometric analysis.

#### von Kossa staining

von Kossa staining was used to detect calcium crystal deposition. Cells were seeded in an eight-well chamber slide system (4 × 10^3^ cells/well), incubated in standard (DMEM/F12, 10% HI-FBS) or osteogenic media for 1, 5, or 15 days, and then washed twice with PBS. They were then fixed in PBS-formalin for 10 min. After washing twice with PBS and once with water, a 2% silver nitrate solution was added. The slides were exposed to UV light for 30 min and, after rinsing once again with water, sodium thiosulphate (5%) was added for 3 min. The slides were again rinsed in water, then Hematoxylin was added for 5 min to counterstain the nuclei. After rinsing in water for the last time, the slides were mounted in glycerol and water solution and then visualized using a Diaplan light microscope (Leitz).

To quantitate the calcium deposition, images were acquired using a Micropublisher 5.0 RTV camera (Q Imaging), and morphometric analysis of the von Kossa staining was performed.

#### Environmental SEM analysis

After seeding in eight-well slides (4 × 10^3^ cells/well) and treatment, cells were washed twice with PBS and fixed in methanol for 10 min. To assess the chemical composition of the cell nodules, environmental SEM (ESEM) analysis with X-ray fluorescence, coupled with energy-dispersive spectroscopy (XRF-EDS), was performed directly on the cells grown on the plastic slides using an ELEMENT instrument (EDAX). This method enables the identification of inorganic compounds within a biological matrix typically comprising carbon, oxygen, and hydrogen. The spectra gathered in the X-ray fluorescence show the peaks of all the elements involved, so a semiquantitative measure of the composition of the inclusions can be obtained by analyzing the net intensities calculated by the peak integral with background line subtraction.

### Detecting and quantitating cell death

#### Cell growth and viability assessment

Cells were plated at 10 × 10^3^ cells/well in 24-well tissue culture plates (Falcon) and grown to 50% confluence in culture medium, then the medium was switched to 1% FBS 24 h before the experiments to induce quiescence. A standard (DMEM/F12, 10% HI-FBS) or osteogenic (NH OsteoDiff medium, Miltenyi Biotec) medium was added to the cells and changed every 2 days. Proliferation was assessed at different times (on days 0, 1, 2, 3, 4, 7, and 8), by means of cell counts and colorimetric assays [24,25]. Briefly, cells were fixed with methanol for 10 min, then stained with 1% Methyl Blue in 0.01 M borate buffer (pH 8.5) for 30 min. After repeated washing, the unbound staining solution was eluted with a 1:1 mixture of ethanol and 0.1 N HCl, and read at an absorbance of 650 nm. Methyl Blue only stains cells attached to the substrate before fixation (i.e. living cells), and thus quantitates their proliferation and viability.

#### Simultaneous annexin V-FITC and propidium iodide staining

Phosphatidylserine externalization was assessed at different time points by measuring annexin V and propidium iodide (PI), using a kit from Affymetrix-eBioscience according to the manufacturer’s instructions. Briefly, after washing with PBS, the cells were detached using trypsin and resuspended at a density of 200–500 × 10^3^ cells/ml in 100 μl annexin-binding buffer (10 mM HEPES, pH 7.4; 140 mM NaCl, and 2.5 mM CaCl_2_) containing 5 μl of annexin V-FITC. This mixture was incubated for 10 min at RT in the dark, then the cells were washed with binding buffer and resuspended in the same buffer containing PI. A minimum of 1 × 10^4^ cells were then analyzed, and the apoptotic stages were examined by flow cytometry using a CytoFLEX cytometer (Beckman Coulter). AnnexinV-positive/PI-negative and annexinV-positive/PI-positive cells were considered in the early and late apoptotic phases, respectively. AnnexinV-negative/PI-positive cells were considered necrotic. All cell populations were counted together and defined as the total dead cell population. Staurosporine (1.0 μM)-treated cells were used as a positive control.

#### Detecting *in situ* cell death with the TUNEL assay

DNA fragmentation was assessed using the terminal deoxynucleotidyl transferase (TdT) mediated dUTP-biotin nick end labeling (TUNEL) assay (TUNEL In Situ Cell Death Detection Kit, Roche). Cells were seeded on chamber slides, and cultured for 1, 5, or 15 days in normal and osteogenic media (4 × 10^3^ cells/well). Cell cultures were fixed with 4% formaldehyde in PBS for 10 min at RT, and permeabilized with 0.1% (vol/vol) Triton X-100 in aqueous 0.1% (wt/vol) sodium citrate for 2 min on ice. Next, the cells were incubated for 1 h at 37°C with a TUNEL reaction mixture comprising a nucleotide mixture in reaction buffer and TdT. The slides were washed three times with PBS, mounted in glycerol and water solution, examined using a DMI600CS-TCS SP8 fluorescence microscope (Leica Microsystems), and analyzed with LAS AF software (Leica Microsystems). Images were acquired using a DFC365FX camera (Leica Microsystems) and morphometric labeling assessment was done. Cells treated with 20.0 U/μl DNase I for 20 min were used as a positive control.

### In-Cell Western

Caspase activation and Runx2 protein expression was assessed using In-Cell Western analysis as described elsewhere [26]. Cells were seeded in a 96-well plate (2 × 10^3^ cells per well) and cultured in standard or osteogenic media for 1, 5, or 15 days. At the end of the treatment, cells were immediately fixed in cold methanol for 10 min at RT, then washed five times with 0.1% Triton X-100 in PBS. The samples were blocked in a solution of 5% milk in PBS containing 0.1% Triton X-100 for 40 min at RT with moderate shaking followed by incubation with specific primary antibodies (Table 1) overnight at 4°C in a humidified chamber. β-tubulin served as an internal control. The plates were washed five times with 0.1% Triton X-100 in PBS and gently agitated for 5 min at RT. Next, secondary antibody (IRDye 800CW donkey anti-Rabbit, 1:800, from LI-COR, Biotechnology, Lincon, NE, U.S.A.) was added to each well and incubated in the dark for 60 min at RT with gentle shaking. Finally, the plates were scanned at 800 nm and intensity of the labeled proteins was measured using the Odyssey Infrared Imaging System (LI-COR). Each experiment was performed in duplicate at least. Negative controls were obtained by omitting the primary antibody during the incubation steps, and background values were obtained by omitting primary and secondary antibodies. The data are shown as the average ± S.D. Staurosporine (1.0 μM)-treated cells were used as a positive control.

**Table 1:** Primary antibodies and dilutions used for In-Cell Western analyses

Target	Clone	Host	Manufacturer	Code	Dilution
Cleaved Caspase-3 (Asp^175^)	5A1E	Rabbit	Cell Signaling Technology	CST-9664	1:1000
Cleaved Caspase-9 (Asp^330^)	D2D4	Rabbit	Cell Signaling Technology	CST-7237	1:1000
Cleaved Caspase-9 (Asp^315^)	-	Rabbit	Cell Signaling Technology	CST-9505	1:1000
Cleaved PARP (Asp^214^)	D64E10	Rabbit	Cell Signaling Technology	CST-5625	1:1000
Runx2/CBFA1	-	Rabbit	Novus Biologicals	NBP1-77461	1:50
β-tubulin	H235	Rabbit	Santa Cruz Biotechnology	sc-9104	1:50

Abbreviation: PARP, poly-(ADP-ribose) polymerase.

### Quantitative real-time PCR

Total RNA was extracted from cell cultures at 1, 5, or 15 days using the RNeasy Mini Kit (Qiagen Limburg, NL) according to the manufacturer’s instructions, and following the spin column protocol. RNA quantity and quality were assessed by spectrophotometric analysis using a NanoDrop ND-1000 (Thermo Fischer Scientific Waltham, MA, U.S.A.), and by capillary electrophoresis using an Agilent 2100 Bioanalyzer (Agilent Technologies Santa Clara, CA, U.S.A.). RNA samples with an A260/A280 ratio between 1.8–2 and an RNA integrity number (RIN) of at least 9 were used for qRT-PCR. A total of 100 ng of total RNA was reverse-transcribed in a final volume of 20 μl containing 5 mM MgCl_2_, 1 mM dNTPs, 2.5 μM random hexamers (Applied Biosystems), 1 U/μl RNAse inhibitor (Applied Biosystems), and 2.5 U/µl MuLV reverse transcriptase (Thermo Fischer Scientific) in a buffer comprising 50 mM KCl and 10 mM Tris/HCl (pH 8.3). Reactions were performed on a 2720 Thermal Cycler (Thermo Fisher Scientific) using the following thermal profile: RT for 10 min, 42°C for 30 min, 65°C for 5 min, and 4°C for 5 min. The primers used are listed in Table 2. Primer pairs for the region of interest were designed using Primer3 software ver. 4.0 (http://primer3.ut.ee), adopting stringent parameters to ensure successful amplification and a convenient experimental design. The National Center for Biotechnology Information (NCBI) Primer-BLAST program was used for *in silico* specificity analysis (www.ncbi.nlm.nih.gov/tools/primer-blast/index.cgi), after which each primer pair was validated. Microchip electrophoresis on an Agilent 2100 Bioanalyzer, Sanger sequencing, and melting curve analyses were used to measure the specificity of the PCRs. Amplification curves were established for all the primers and resulted in an 85% efficiency, at least. qRT-PCR was performed using an iCycler Thermal Cycler (Bio–Rad, Hercules, CA, U.S.A.) and SYBR Green I technology with iQ™ SYBR Green Master Mix (Bio–Rad) in a final volume of 20 µl final volume containing 1 µl of reverse-transcribed cDNA template. An appropriate primer concentration (0.3 µM) was used, and the annealing temperatures are listed in Table 1. Data analysis was performed using the ΔΔ*C*_t_ method, normalizing the data to two different housekeeping genes (glyceraldehyde 3-phosphate dehydrogenase (*GAPDH*) and hypoxanthine guanine phosphoribosyl transferase (*HPRT1*)) according to the minimum information for publication of quantitative real-time PCR experiment (MIQE) guidelines [27]. The normalized relative quantitation (nRQ) was calculated as 2^−ΔΔ*C*^_t_. A melting curve analysis was performed to identify any non-specific amplification products. The results were obtained from two separate experiments performed in triplicate.

**Table 2:** Primer sequences used in qRT-PCR analyses

Primers	Nucleotide sequence	T annealing (°C)	NCBI reference sequence	Efficiency (%)
GAPDH Fw	GAAGGTGAAGGTCGGAGT	60	NM_17851.1	99.0
GAPDH Rev	TGGCAACAATATCCACTTTACCA			
HPRT1 Fw	CCTGGCGTCGTCATTAGTGA	60	NM_000194.2	97.8
HPRT1 Rev	TCTCGAGCAAGACGTTCAGT			
Osteonectin Fw	CCTGGATCTTCTTTCTCCTTTGC	60	NM_001309443.1	97.9
Osteonectin Rev	ATCAGGCAGGGCTTCTTGCT			
Osteopontin Fw	CGAGACCTGACATCCAGTACC	62	NM_001251830.1	96.2
Osteopontin Rev	GATGGCCTTGTATGCACCATTC			
Runx2 Fw	CATTTCAGATGATGACACTGCC	62	NM_001024630.3	95.2
Runx2 Rev	GGATGAAATGCTTGGGAACTG			
BAX Fw	GCCGTGGACACAGACTCC	60	NM_001291428.1	91.2
BAX Rev	AAGTAGAAAAGGGCGAAACC			
BCL2 Fw	TCATGTGTGTGGAGAGCGTCAA	60	NM_000633.2	88.9
BCL2 Rev	CAGCCAGGAGAAATCAAACAGAGG			
GDNF Fw	GGCTATGAAACCAAGGAGGAACTG	64	NM_000514	98.7
GDNF Rev	TCCACCACCCTGTTGCTGTA			

### Morphometric analysis

Morphometric analysis was performed by ImagePro Plus software (Media Cybernetics). For each experimental sample, a maximum of 15 images at 200× or 400× magnification was analyzed. Signals were acquired for all the images with the same brightness and contrast characteristics from three different slides and the quantity was expressed as the percentage of the mean area covered by pixels [28,29].

### Statistical analysis

Data are presented as means ± S.D. Multiple group means were compared using ANOVA with a between-within design and Bonferroni’s correction. Data from the morphometric analysis were examined using a non-parametric test (the Mann–Whitney U test), and statistical significance was established with the Primer software (McGraw-Hill). A *P*-value of less than 0.05 was considered statistically significant.

## Results

### An osteogenesis-like process occurred in the calcification of *GDNF*-silenced HK-2 cells

**Figure 1:**
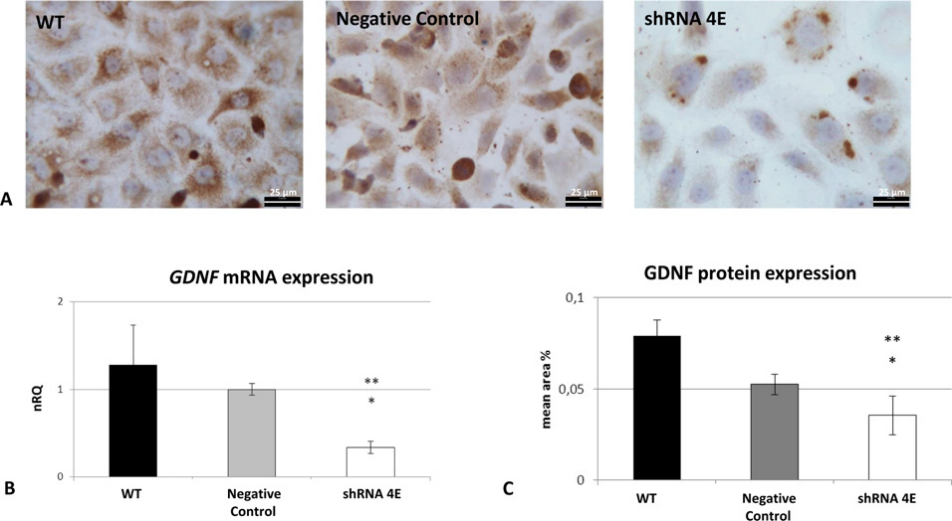
*GDNF* knockdown in an HK-2 cell line (**A**) GDNF protein detected using ICC in WT, Negative Control, and silenced (shRNA 4E) cells (Diaplan light microscope (Leitz), magnification: 400×). GDNF immunostaining with a polyclonal anti-GDNF antibody revealed a lower signal in the shRNA 4E than in either of controls (WT and Negative Control cells). Light microscope images are representative of the results of two independent experiments. Bar =25 µm. (**B**). Changes in *GDNF* gene expression as determined by qRT-PCR (ΔΔ*C*_t_ method) in WT, negative control, and shRNA 4E cells. (**C**) Changes in GDNF protein expression as determined by morphometric analysis. The results are presented as the mean ± S.D. of two independent experiments performed in triplicate. Statistically significant differences between the shRNA 4E and the WT (*P<0.005) and Negative Control (**P<0.05) cells were analyzed using ANOVA with a between-within design and Bonferroni’s correction.

**Figure 2:**
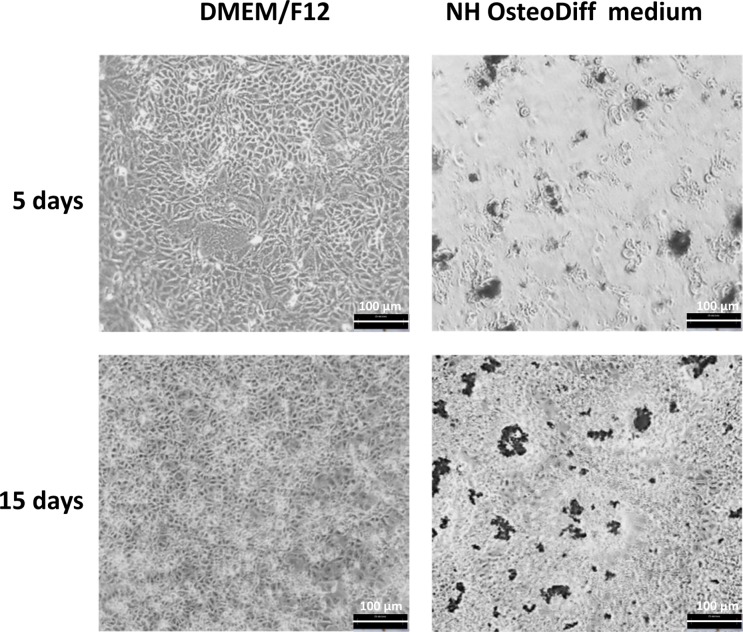
Phase-contrast inverted microscope images of silenced cells (shRNA 4E) grown in DMEM/F12 supplemented with 10% HI-FBS or NH OsteoDiff medium shRNA 4E cells grown in osteogenic medium exhibited multilayer growth with cells retracting from some areas and grouping into multicellular aggregates or nodules with abundant dense deposits. The images (acquired with Hund Wetzlar, Wilovert, magnification: 200×) are representative of two independent experiments. Bar =100 µm.

In *GDNF*-silenced HK-2 cells, gene knockdown resulted in an approximately 70% reduction in *GDNF* transcript and 60% in protein levels (Figure 1). There was a multilayered growth in both the control and the silenced cells cultured in osteogenic conditions, with cells retracting from some areas and grouping into multicellular aggregates or nodules. The silenced cells also exhibited many more nodules with dense deposits than the control cells, and this became increasingly evident over time (Figure 2).

**Figure 3:**
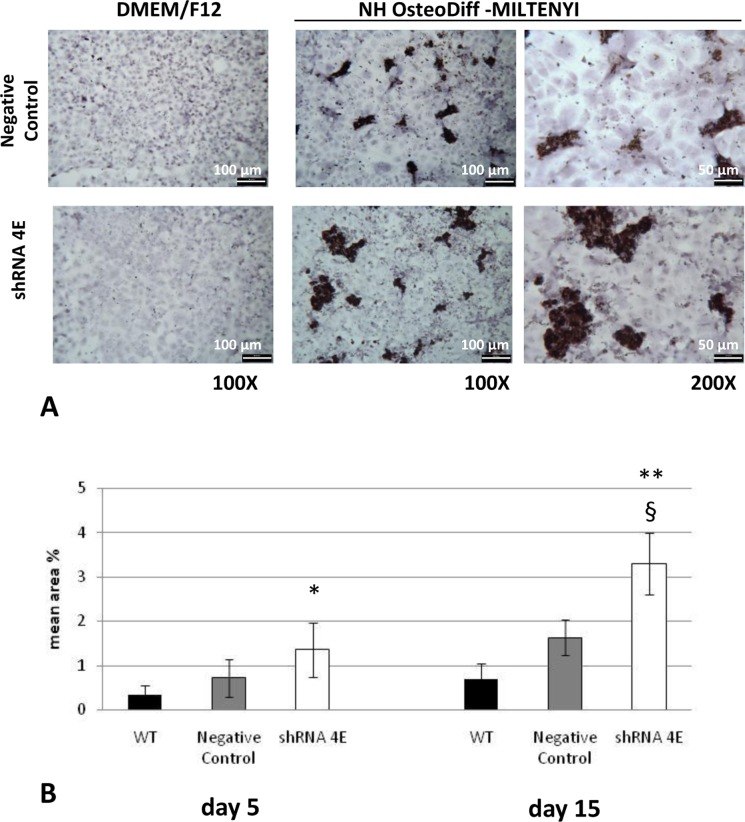
Calcium detection in HK-2 cells cultured in either DMEM/F12 supplemented with 10% HI-FBS or NH OsteoDiff medium (**A**) Light microscope images (Diaplan light microscope (Leitz), magnification: 100–200×) of von Kossa staining revealed calcium deposits in some cells and nodules in the Negative Control and silenced (shRNA 4E) cells grown in osteogenic medium for 15 days. No calcium deposition was seen in Negative Control or silenced cells cultured under standard conditions (DMEM/F12 supplemented with 10% HI-FBS). Low magnification images showed much more abundant dark deposits in the silenced than in the Negative Control cells. The images are representative of two independent experiments. Bars =100 µm (left) and 50 µm (right). (**B**) Quantitative analysis of von Kossa staining with morphometric analyses on days 5 and 15. Statistically significant differences emerged between the shRNA 4E cells and the WT and Negative Control cells at 5 (*P<0.05) and 15 days (**P<0.005 shRNA 4E compared with WT and ^§^*P*<0.01 compared with Negative Control) using a non-parametric test (Mann–Whitney U test) and Primer software (McGraw-Hill).

**Figure 4:**
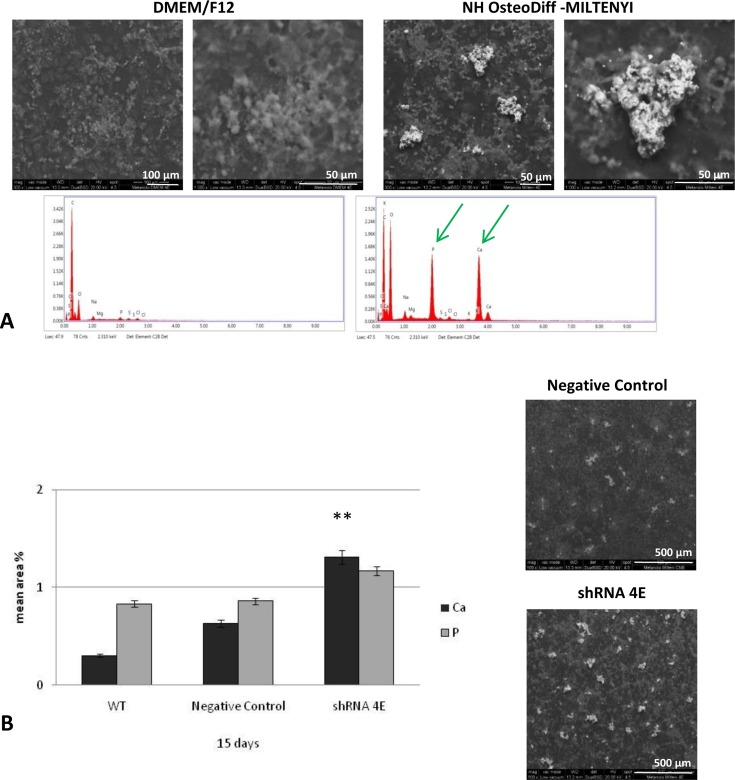
ESEM analysis of HK-2 cells cultured in DMEM/F12 supplemented with 10% HI-FBS or NH OsteoDiff medium (**A**) In silenced cells, ESEM images and spectra confirmed the presence of calcium (Ca) and phosphate (P) in the nodules on day 15 (green arrow). No calcium-phosphate (Ca_2_PO_4_) deposits were seen in silenced cells cultured under standard conditions. The images are representative of two independent experiments. Bars =100 µm (left), 50 µm (right). (**B**) Quantitative analysis of calcium and phosphate levels in WT, Negative Control, and shRNA 4E cells grown in NH OsteoDiff medium for 15 days. Bar =500 µm. Statistically significant differences emerged using a non-parametric test (Mann–Whitney U test), and Primer software (McGraw-Hill) (**P<0.005 shRNA compared with Negative Control and WT).

Notably, this distinct ‘hill and valley’ morphology developed in parallel with the deposition of calcium aggregates (as revealed by von Kossa staining), which was detectable already after 5 days of culture, and increased substantially over 15 days (Figure 3). When the von Kossa staining was measured using morphometric analysis, there was significantly more calcium deposition in the silenced than in the control cells at 5 (*P*<0.05) and 15 days (*P*<0.005 compared with WT; *P*<0.01 compared with negative control) (Figure 3B). No calcium deposition was seen in the control or silenced cells cultured under standard conditions (Figure 3A). ESEM analysis revealed that the granular concretions ranged from 1.0 to 40 μm in diameter, and contained an abundance of calcium and phosphate (Figure 4). The concomitant presence of calcium and phosphate suggests Ca_2_PO_4_ precipitation in the nodules. Much smaller amounts of these aggregates were also seen in the WT and control cells cultured in osteogenic conditions (Figure 4B).

**Figure 5:**
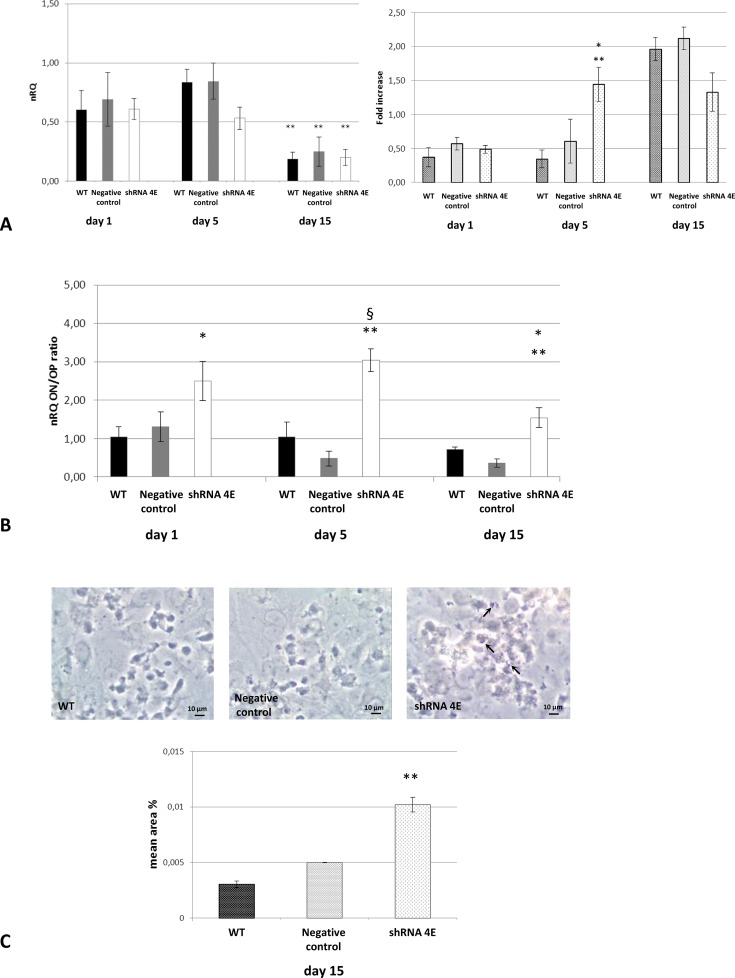
Osteogenic-like process in WT, Negative Control, and silenced (shRNA 4E) cells grown in osteogenic NH OsteoDiff medium (**A**) Left: expression of *Runx2* gene by qRT-PCR (ΔΔ*C*_t_ method); right: expression of Runx2 protein by ICW Fluorescence quantitation at 800 nm normalized to the β-tubulin signal. (*P<0.05; **P<0.005 shRNA compared with Negative Control and WT). (**B**) Expression of osteogenesis-related genes as determined by qRT-PCR (ΔΔ*C*_t_ method). Results are expressed as the ratio of nRQ osteonectin (ON) to nRQ osteopontin (OP) (^§^*P*<0.01, shRNA 4E compared with WT; *P<0.05, shRNA 4E compared with WT; **P<0.005, shRNA 4E compared with Negative Control). (**C**) ALP staining quantitation by morphometric analysis of light microscope images (Leica DMIL LED, magnification: 400×) (**P<0.005 shRNA compared with Negative Control and WT). ALP appears as an intense blue/purple stain in the cells (arrows). The images are representative of two independent experiments. Bar =10 µm. Results are presented as the mean ± S.D. of two independent experiments performed in triplicate. Statistically significant differences emerged using ANOVA with a between-within design and Bonferroni’s correction.

The gene expression of osteoblastic markers such as Runx2 (an early osteogenic programming gene) and osteopontin and osteonectin (later osteogenic programming genes), was measured over the course of osteogenic induction to see if Ca_2_PO_4_ deposition was related to an osteogenic-like process. We detected a significant increase in Runx2 expression at days 1 and 5 compared with day 15 (Figure 5A). However, no difference between *GDNF*-silenced HK-2 cells and control cells was found. A difference between control and silenced cells was instead demonstrated by evaluating Runx2 expression at protein level by In-Cell Western. In fact, Runx2 was significantly higher in silenced cells than in control cells at 5 days (*P*<0.05 compared with negative control *P*<0.005 compared with WT).

Figure 5B shows that the osteonectin/osteopontin ratio was significantly higher in the *GDNF*-silenced HK-2 cells than in the control cells. The highest osteonectin/osteopontin ratio was recorded after 5 days (*P*<0.005 shRNA 4E compared with negative control; *P*<0.01 shRNA 4E compared with WT).

On cytochemical examination of ALP activity, *GDNF*-silenced cells showed a significant higher positive signal than control cells after 15 days of treatment with osteogenic medium (*P*<0.005 compared with negative control and WT) (Figure 5C). Positive signals were detected predominantly around the nodules and were observed also in some of the control cells.

### Massive cell death occurred in the calcified nodules of *GDNF*-silenced HK-2 cells

**Figure 6:**
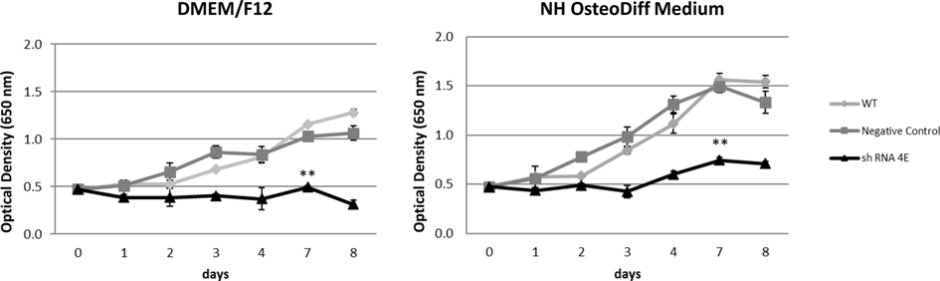
Cell proliferation and viability Results of Methyl Blue assay (based on optical density) of WT, Negative Control, and silenced (shRNA 4E) cell growth in standard (DMEM/F12 supplemented with 10% HI-FBS) and osteogenic (NH OsteoDiff) conditions. Data are presented as the mean ± S.D. of three independent experiments. Statistically significant differences emerged between the shRNA 4E and the WT and Negative Control cells (**P<0.005) using ANOVA with a between-within design and Bonferroni’s correction.

Cell proliferation was analyzed first. Cells were monitored for 1–8 days, and control cells exhibited a similar growth in the standard and osteogenic media, with a gradual time-dependent increase in their growth (Figure 6). At each time point on the growth curve, *GDNF*-silenced HK-2 cells exhibited significantly less proliferation than control cells (*P*<0.005), pointing to the role of GDNF as a survival factor for renal tubular cells too. The silenced cells in osteogenic medium did start to grow after 4–7 days of culture, however, albeit more slowly than the control cells (*P*<0.005) (Figure 6).

**Figure 7:**
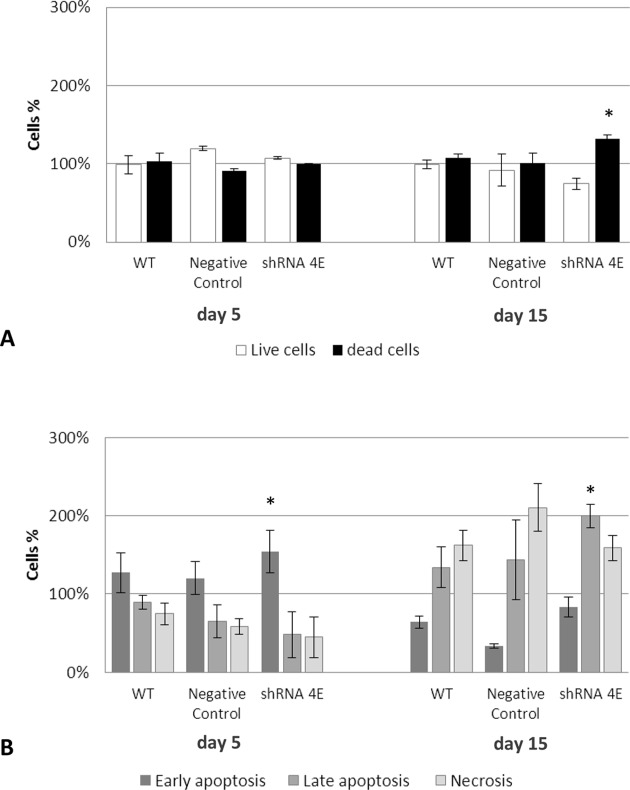
Double staining with annexin V and PI in WT, Negative Control, and silenced (shRNA 4E) cells grown in osteogenic (NH OsteoDiff) medium for 5 and 15 days (**A**) Percentages of dead and live cells. (**B**) Cell population (necrosis, early and late apoptosis) involved in the death process. Results are presented as the mean ± S.D. of two independent experiments performed in triplicate. Statistically significant differences emerged using ANOVA with a between-within design and Bonferroni’s correction (*P<0.05 shRNA 4E compared with Negative Control and WT).

Then the cell death process was examined and quantitated by means of a simultaneous staining with annexin V-FITC and PI, which enabled us to discriminate between apoptotic and non-apoptotic cell death. Under osteogenic conditions, there was an overall increase in the cell death rate at 15 days compared with the rate at 5 days, in both control and silenced cells (Figure 7A), but the difference was greater in the latter (*P*<0.05). Phosphatidylserine externalization usually occurs in the inner leaflet of the plasma membrane, and is one of the earliest signs of apoptosis, preceding DNA fragmentation and membrane blebbing. After 5 days in osteogenic medium, there was a predominant population in the early apoptotic phase (annexinV-positive/PI-negative), which was significantly greater in the silenced than in the control cells (*P*<0.05). (Figure 7B). After 15 days in osteogenic medium, there were two predominant types of dead cell population, one only PI-positive (necrotic cells), the other was annexinV-positive and PI-positive (cells in late apoptosis). This latter population was significantly more evident in the silenced than in the control cells (*P*<0.05) (Figure 7B). The various phases of the process appeared to have been completed within the time window considered, indicating a transition from early to advanced or late apoptosis. In standard culture conditions, the dead cells were more numerous amongst the silenced cells, but they were mainly in the early phase of apoptosis (results not shown). These results again point to GDNF acting as a survival factor for the renal tubular cells.

**Figure 8:**
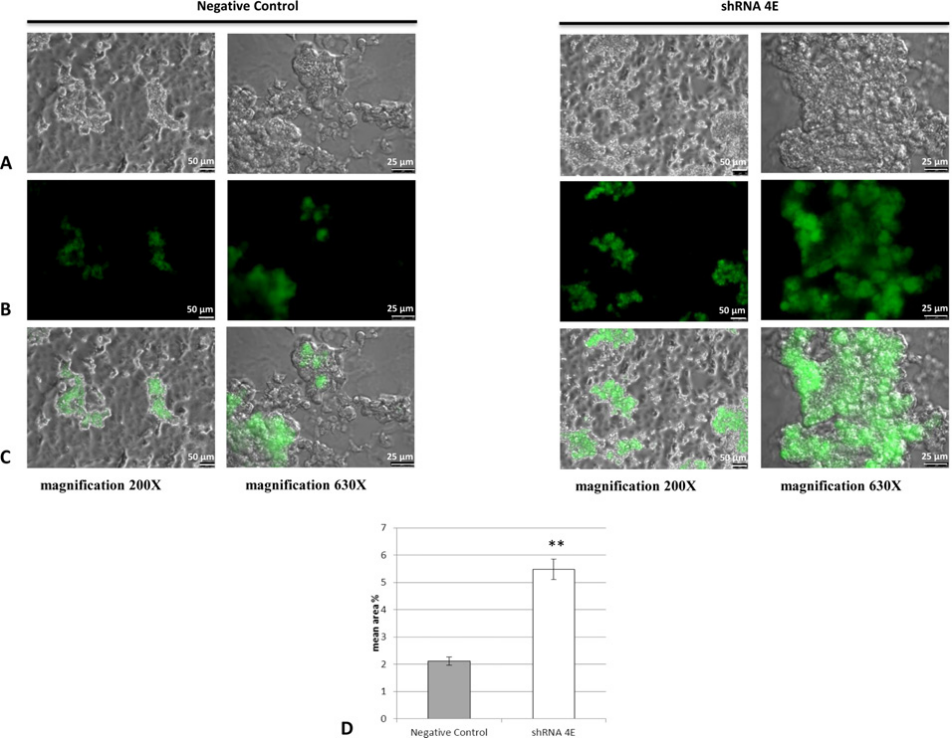
Immunofluorescence images of TUNEL-stained apoptotic nuclei (green staining) in Negative Control and silenced (shRNA 4E) cells grown in NH OsteoDiff medium for 15 days The images (acquired using DMI600CS-TCS SP8 fluorescence microscope) are representative of two independent experiments. (**A**) Bright field; (**B**) FITC; (**C**) merge. Bars =50 and 25 µm. (**D**) Quantitative analysis of TUNEL staining using morphometric analysis. Statistically significant differences emerged between the shRNA 4E cells and Negative Control cells using with a non-parametric test (Mann–Whitney U test) and Primer software (McGraw-Hill) (**P<0.005).

One of the most common hallmarks of apoptosis is DNA fragmentation, so DNA content in the *GDNF*-silenced HK-2 cells was analyzed by TUNEL assay. While no positive staining was observed at 5 days, several nodules had positively stained nuclei in the silenced cells after 15 days of culturing, meaning that these cells were probably apoptotic (Figure 8). There were few positive nodules in the control cells but the signal was less intense, and the number of late apoptotic cells was significantly lower than in the silenced cells (*P*<0.05), while there were more necrotic cells (Figure 7B). No staining was apparent in any areas/cells around the nodules or in cells cultured in standard conditions (results not shown). Taken together, these findings indicate that the presence of apoptotic bodies in the nodules was associated with the calcification process.

### The death process in *GDNF*-silenced HK-2 cells is caspase independent

**Figure 9:**
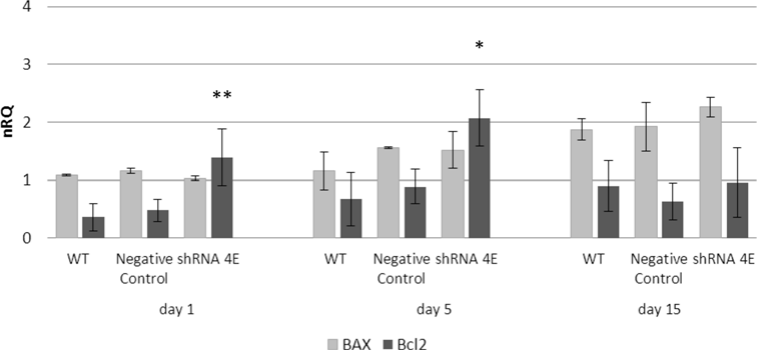
Expression of apoptosis-related genes in WT, Negative Control, and silenced (shRNA 4E) cells grown in NH OsteoDiff medium Changes in *BCL2* and *BAX* mRNA levels measured using qRT-PCR (ΔΔ*C*_t_). Results are presented as the mean ± S.D. of two independent experiments performed in triplicate. Statistically significant differences were calculated using ANOVA with a between-within design and Bonferroni’s correction (*P<0.05; **P<0.005 shRNA compared with Negative Control and WT).

Activation of the apoptotic process was examined by measuring *BCL2* (anti-apoptotic) and *BAX* (pro-apoptotic) gene expression using qRT-PCR. Silenced cells cultured in the osteogenic medium showed a significantly higher *BCL2* expression at 1 (*P*<0.005) and 5 (*P*<0.05) days than the control cells, while *BAX* expression increased over time but no more than in the control cells (Figure 9).

**Figure 10:**
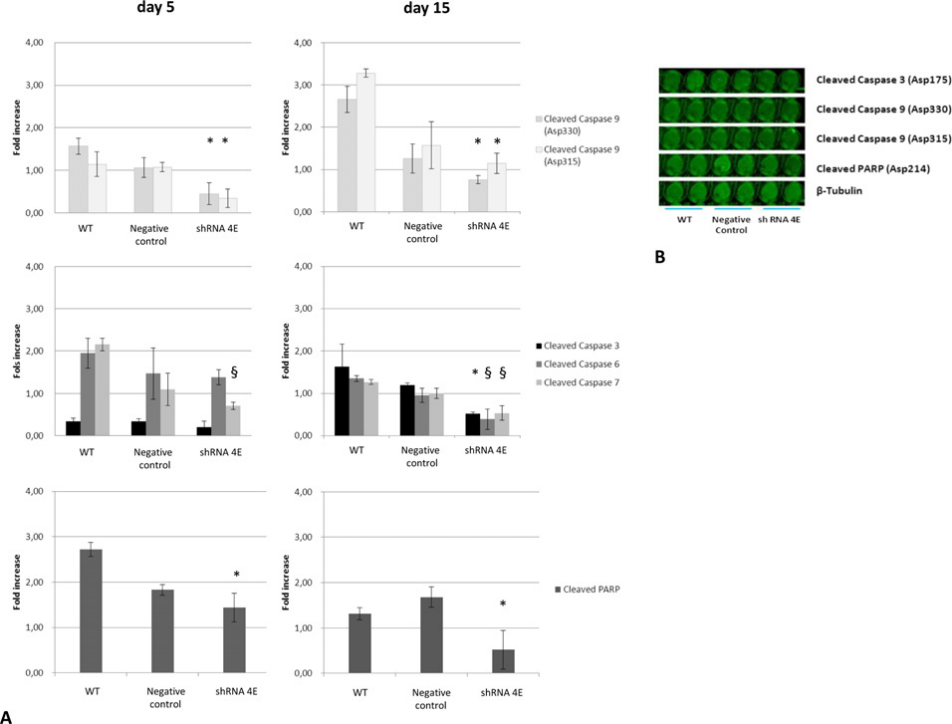
Caspase activation in WT, Negative Control, and silenced (shRNA 4E) cells grown in NH OsteoDiff medium (**A**) Fluorescence quantitated at 800 nm and normalized to the β-tubulin signal. Results are presented as the mean ± S.D. of two independent experiments performed in triplicate. Statistically significant differences emerged using ANOVA with a between-within design and Bonferroni’s correction (*P<0.05; ^§^*P*<0.005 shRNA compared with Negative Control and WT). (**B**) Representative image acquired at a wavelength of 800 nm.

The onset of apoptosis is characterized by caspase-3, -6, -7, and -9 activation in the cytosol. In-Cell Western analysis was performed on the control and *GDNF*-silenced cells grown in osteogenic medium to detect any presence of activated caspases. The levels of cleaved caspase-9 (an initiator caspase), and cleaved caspase-3, -6, and -7 (effector caspases) were measured, and so were those of cleaved nuclear protein poly-(ADP-ribose) polymerase (PARP), which is a caspase substrate. Silenced cells exhibited significantly lower caspase and PARP levels than control cells at both 5 and 15 days (Figure 10A).

## Discussion

We previously reported that an unexpected calcification process spontaneously occurred in primary papillary cells obtained from the kidney biopsy of a patient with MSK and interstitial nephrocalcinosis who harbors a *GDNF* gene mutation [8]. We also demonstrated that culturing *GDNF-*silenced HK-2 cells in osteogenic medium induced Ca_2_PO_4_ deposition and the expression of osteoblast differentiation markers [8]. Further investigations were warranted, however, to see whether *GDNF* down-regulation directly causes or contributes to the osteogenic-like calcification process seen in primary renal and HK-2 cells.

The present study sought to ascertain whether down-regulating *GDNF* could lead to cell death and thus trigger a process of nephrocalcinosis under certain conditions. GDNF is known to have survival-supporting properties in neurones [30]. In the kidney, it was found to act in an autocrine manner, supporting podocyte survival [31]. The important protective role of GDNF in the apoptotic process is supported by the literature regarding not only the kidney [31–33], but also the bowel. In Crohn’s disease, for instance GDNF protects the enteric glial cells of the intestinal mucosa against apoptosis, thus preserving its integrity and function [34]. *GDNF* down-regulation might therefore conceivably trigger calcification by favoring cell death.

A previously adopted *in vitro* experimental model of *GDNF* down-regulation in HK-2 cells was used here to elucidate the mechanisms underlying the calcification process in renal cells [8]. *GDNF* expression levels were monitored at various times during osteogenic induction and under standard conditions, and the results indicated that *GDNF* expression was always significantly lower in silenced than in control cells (results not shown).

The occurrence of an osteogenesis-like process in our model, which began after 5 days of culture, was confirmed by the early Runx2 up-regulation and by the increase in the osteonectin/osteopontin expression ratio. This observation is in keeping with findings suggesting Runx2 triggers the expression of major bone matrix genes during early stages of osteoblasts differentiation, and its overexpression stops in the later stage and is not essential for maintenance of these gene expressions in mature osteoblasts [35,36]. Osteonectin is known to promote the formation of mineral deposits [37], whereas osteopontin is considered a powerful inhibitor of crystal formation [38–40]. Our results indicate that the balance between these latter pro- and anti-osteogenic factors in the *GDNF*-silenced cells favored an osteogenic process. The quantitation of ALP activity, a later osteoblast differentiation marker [41], further confirmed the osteoblast-like phenotype of silenced cells cultured in osteogenic medium. Thus, the pattern of expression of both Runx2, and osteonectin/osteopontin, as well as ALP confirm that an active osteogenic-like process occurred in the *GDNF*-silenced HK-2 cells in osteogenic culture, inducing calcification that was detectable already after 5 days of culture, and increased considerably over 15 days. This calcification was localized exclusively in the nodules and/or cell aggregates. These results further support our hypothesis of a procalcifying stimulus of *GDNF* down-regulation [8].

Having ascertained that GDNF massively favored the Ca_2_PO_4_ deposition process in our *in vitro* model of nephrocalcinosis, we investigated whether cell death had occurred under the same conditions. The slow proliferation of silenced cells in the standard and osteogenic media appeared to support the primary hypothesis of this work, i.e. that *GDNF* down-regulation may be related to cell death phenomena, and potentially to apoptosis. Different methods were used to investigate this issue, which shed light on how the cell death process occurred.

Both late apoptotic and necrotic cells were amply represented in the control and silenced cells cultured in osteogenic medium for 15 days. In the *GDNF*-silenced cell population, the apoptotic cells were found in abundance and exclusively in the nodules where calcium and phosphate precipitated, suggesting that they served as nuclei for calcification.

The increase in annexin V-positive cells at 15 days, associated with PI-positive staining, might be not only due to the phosphatidylserine externalization associated with late phases of apoptosis, but also due to the loss of membrane integrity characteristic of secondary necrosis (i.e. necrosis after apoptosis). In fact, cells with impaired membranes are also labeled with annexin V-FITC, which enters the cells and stains the internal surface of the plasma membrane, where phosphatidylserine is normally found. Some authors therefore suggest that concurrently assessing both probes only enables us to distinguish between early-stage apoptotic cells (annexinV-positive/PI-negative) and necrotic cells (annexinV-positive/PI-positive, and annexinV-negative/PI-positive) [42,43]. In other words, the annexinV-positive/ PI-positive cells may be in a later stage of apoptosis and/or undergoing secondary necrosis.

Necrosis causes DNA cleavage too, though such cleavage is not characteristically internucleosomal. TUNEL assay may not distinguish the internucleosomal DNA cleavage of apoptosis from the DNA cleavage of necrosis [44,45]. The present results nonetheless confirm that many cells within the nodule underwent cell death after between 5 and 15 days of osteogenic culture. They also suggest that both necrotic and apoptotic cells may serve as nuclei for calcification. In fact, the osteogenic culture of *GDNF*-silenced cells induced cell death in a time-dependent manner, and the apoptotic process was completed by day 15, when the quantity of calcified nodules was at its highest.

During the apoptotic process, mitochondrial outer membrane permeabilization is regulated by various proteins, such as those encoded by the mammalian *BCL2* family of genes [46,47], which can directly promote or inhibit apoptosis. BCL2 protein inhibits the formation of a pore formed by BAX, which promotes apoptosis by competing with BCL2. *BAX* and *BCL2* were therefore selected as pro- and anti-apoptotic markers, respectively, for our gene expression study. While a temporal increase in *BAX* was observed under osteogenic conditions (with no difference between the silenced and control cells), there was a surprisingly and significantly higher level of *BCL2* in the *GDNF*-silenced cells after 1 and 5 days of culture. BCL2 is an anti-apoptotic protein that regulates apoptosis at mitochondrial level by maintaining the integrity of the mitochondrial membrane and blocking the release of apoptosis factors such as cytochrome *c* from the mitochondria to the cytoplasm, thereby preventing the activation of the caspase cascade. Consistently, we found a lower quantity of cleaved caspases in the *GDNF*-silenced cells than in the control cells. The high *BCL2* expression in the former cells probably inhibits caspase cleavage and subsequent activation.

These findings in the *GDNF*-silenced cells appear to contrast with the flow cytometry and TUNEL results, which confirmed a massive presence of cells in the late phase of apoptosis. Though they were undergoing apoptosis, the control cells cultured in osteogenic conditions did not display *BCL2* up-regulation or caspase down-regulation, indicating that the apoptotic process underway was caspase dependent. It is worth adding that calcification was less pronounced in the control cells that underwent a quantitatively and qualitatively different cell death process: a caspase-independent cell death seemed to be peculiar to *GDNF*-silenced cell calcification. These cells also underwent a process of necrosis. Collectively, these data seem to indicate that – instead of preventing cell death as such – inhibiting postmitochondrial protease activation causes a shift from the apoptotic to another mode of programmed cell death.

In an *in vivo* model of vascular calcification, cell death proved to be caspase-dependent apoptosis [48]. During osteoblastic differentiation of human mesenchymal cells *in vitro*, both necrotic and apoptotic cells were found to serve as nuclei for calcification [49]. In our *in vitro* model of renal calcification, the cell death process was caspase independent. Several types of programmed cell death in which caspase activation plays no part [50,51] have been described so far, including: those that involve no DNA fragmentation, such as autophagy-related cell death [52] and paraptosis [53,54]; and those that lead to a regulated form of necrosis or necroptosis [55,56], which is considered an adaptive response to ensure the elimination of damaged cells and protect the well-being of the organism as a whole. Necroptosis is activated in a programmed fashion similar to apoptosis, but morphologically exhibits the hallmarks of necrosis. The programmed cell death seen in our model could be classified as necroptosis, as its characteristics were intermediate between apoptosis and necrosis. The cell death seen in our model was clearly caspase independent, however. Several necroptotic pathways have recently been discovered in renal cells, depending on the initial stimulus, such as calcium crystals [57], cadmium [58], and TNF-α [59], or on the type of acute kidney injury [60–62].

Our evidence of the effect of *GDNF* silencing on cell death is consistent with reports from other authors [63,64], who found no mitochondrial pathway activation in GDNF-deprived sympathetic neurones. Cytochrome *c* was not released from the mitochondria into the cytosol; and BAX and caspases-9 and -3 were not involved in the cell death process. Many cells in which the main mitochondrial death pathway are genetically or pharmacologically disabled can still die via an alternative pathway, which is often caspase independent [53,64–68].

The undeniable complexity of the mechanisms involved in cell death has shown that, under certain conditions, markers of apoptosis and necrosis may be found simultaneously, indicating that more than one cell death mechanism can be activated at the same time [69]. While there may be signs of different cell death pathways being involved, the fastest and most effective pathway is often predominant [70]. In support of our data, studies focussing on the process of osteoblast maturation (a typical osteogenic process) have provided no direct evidence of the activation of caspase-dependent apoptotic processes accompanying osteogenesis [71–73].

In conclusion, GDNF was confirmed as an adaptive survival factor, and its alteration appears to have a key role in nephrocalcinosis. The findings emerging from the present study indicate that *GDNF* down-regulation can trigger apoptosis in human renal tubular cells and, under certain environmental conditions (e.g. in an osteogenic medium), this can strongly facilitate calcium-phosphate deposition. Our results also suggest, however, that this apoptotic process is caspase independent. Further studies are needed to better define the necroptosis signaling underlying the observed programmed cell death phenomenon.

We are tempted to speculate that, if cell death is an important event in the pathogenesis of renal ectopic calcification, any damage that shifts the balance between cell survival and cell death toward the latter could lead (in conjunction with a particular renal milieu) to the phenomenon of interstitial nephrocalcinosis.

It has long been known that ectopic calcification follows necrosis. In the kidney, cortical nephrocalcinosis (a rare condition usually resulting from severe destructive disease in the cortex) has been attributed to the presence of necrotic tubular cells [18]. To our knowledge, no attention has been paid as yet to the role of cell death in the more common interstitial nephrocalcinosis.

## Clinical perspectives

We know that ectopic calcification may follow necrosis. In the kidney, cortical nephrocalcinosis (a rare condition usually resulting from severe destructive disease in the cortex) has been attributed to the presence of necrotic tubular cells. To our knowledge, no attention has been paid as yet to the role of cell death in the more common interstitial nephrocalcinosis. In the present study, we demonstrated that programmed cell death played an important part in the renal cell calcification process in an *in vitro* model of nephrocalcinosis. We speculate that, if cell death is an important event in the pathogenesis of renal calcification, any damage that shifts the balance between cell survival and cell death toward the latter could lead (in conjunction with a particular renal milieu) to the phenomenon of interstitial nephrocalcinosis. The discovery of alternative, caspase-independent cell death pathways demands the search for new strategies – not only for treating disorders such as cancer or ischemic and degenerative diseases, but also for nephrocalcinosis and/or nephrolithiasis.

## Abbreviations

ALP: alkaline phosphatase
DMEM/F12: Ham’s F12 and Dulbecco’s modified Eagle’s growth medium
ESEM: environmental SEM
GDNF: glial cell-derived neurotrophic factor
HI-FBS: heat-inactivated FBS
HK-2: human kidney-2
ICC: immunocytochemistry
MSK: medullary sponge kidney
MuLV: murine leukemia virus
PARP: poly-(ADP-ribose) polymerase
PI: propidium iodide
qRT-PCR: quantitative real-time PCR
RT: room temperature
Runx2: Runt related transcription factor 2
TdT: terminal deoxynucleotidyl transferase
TNF: tumor necrosis factor
TUNEL: TdT dUTP-biotin nick end labeling
VSMC: vascular smooth muscle cell
WT: wild-type
